# Thermal and Current Flow Effects of a Capacitive–Resistive Electric Transfer Application Protocol on Chronic Elbow Tendinopathy. A Cadaveric Study

**DOI:** 10.3390/ijerph18031012

**Published:** 2021-01-24

**Authors:** Carlos López-de-Celis, Jacobo Rodríguez-Sanz, César Hidalgo-García, Simón A. Cedeño-Bermúdez, Daniel Zegarra-Chávez, Pablo Fanlo-Mazas, Albert Pérez-Bellmunt

**Affiliations:** 1 ACTIUM Functional Anatomy Group, 08195 Barcelona, Spain; carlesldc@uic.es (C.L.-d.-C.); jrodriguezs@uic.es (J.R.-S.); simoncedeno@uic.es (S.A.C.-B.); dzegarra@uic.es (D.Z.-C.); 2 Faculty of Medicine and Health Sciences, Universitat Internacional de Catalunya, 08195 Barcelona, Spain; 3 Fundació Institut Universitari per a la Recerca a l’Atenció Primària de Salut Jordi Gol i Gurina (IDIAPJGol), 08007 Barcelona, Spain; 4 Facultad de Ciencias de la Salud, Unidad de Investigación en Fisioterapia, Universidad de Zaragoza, C/Domingo Miral S/N, 50009 Zaragoza, Spain; hidalgo@unizar.es (C.H.-G.); pfanlo@unizar.es (P.F.-M.)

**Keywords:** tennis elbow, cadaver, diathermy, physical therapy

## Abstract

Lateral elbow tendinopathy, or “tennis elbow,” is a pathology that affects around 1.3% of the general population. Capacitive–resistive electric transfer therapy aims to provoke temperature and current flow changes in superficial and deep tissues. The aim of this in vitro study was to analyze the thermal behavior and transmission of electric current on the superficial and deep tissues of the elbow during the application of different modalities of a capacitive–resistive electric transfer treatment protocol for chronic elbow tendinopathy. A cross-sectional study was designed; five fresh cryopreserved cadavers (10 elbows) were included in this study. A 30 min intervention was performed based on a protocol commonly used in clinics for the treatment of chronic lateral elbow tendinopathy by diathermy using the “T-Plus.” Common extensor tendon, radiohumeral capsule, and superficial temperatures were registered after each application for the duration of the 30 min treatment protocol. During all applications, we observed a current flow of over 0.03 A. The protocol showed a statistically significant increase in superficial temperature by 24% (5.02°) (*p* < 0.005), the common extensor tendon by 19.7% (4.36°) (*p* < 0.007), and the radiohumeral joint capsule by 17.5% (3.41°) (*p* < 0.005) at the end of the 30 min protocol compared with the baseline temperature. The different applications of the protocol showed specific effects on the temperature and current flow in the common extensor tendon and radiohumeral capsule. All applications of the protocol produced a current flow that is associated with the generation of cell proliferation. These results strengthen the hypothesis of cell proliferation and thermal changes in deep and distal structures. More studies are needed to confirm these results.

## 1. Introduction

Lateral elbow tendinopathy, or “tennis elbow,” is a pathology that affects around 1.3% of the general population, with equal affectation in men and women [1]. People in manual labor, those who use vibration tools, and throwing athletes have a greater risk of suffering this pathology [2]. Lateral elbow tendinopathy is primarily caused by repeated stress in the extensor tendon, in particular the extensor carpi radialis brevis, although it can also be caused by direct traumatism or overstretching [2,3]. Although the pathophysiological mechanism of tendinopathy has not been elucidated, it is believed that chronic tendinopathy is produced by a degenerative mechanism of the extensor tendon. This happens through hypoxia and tendon fibrosis, which could lead to the formation of calcium deposits [3]. As a result of this tissue hypoxia, fibrosis and the liberation of algesic substances occur, causing pain and muscle spasm [4,5].

Vascular supply is an important component in the repair of the tendon tissue [6]. Studies performed in animals have shown that the interruption of vascular supply in the tendon produces changes, for instance, in the fascicles of the tendon or in the collagen strands. These adaptations caused by the change of vascular supply in an animal’s tendon are similar to those observed in human chronic tendinopathy [7,8].

For this reason, different rehabilitation techniques attempt to increase the blood supply of the musculotendinous tissue by increasing the temperature. Different studies have shown that increasing the temperature by 1 °C also increases the nerve conduction velocity, the enzymatic action, and the release of oxyhemoglobin [9,10,11,12].

Capacitive–resistive energy transfer (CRet) therapy is a noninvasive electrothermal therapy classified as deep thermotherapy, which is based on the application of an electric current in the radio frequency range of 300 kHz–1.2 MHz. This type of therapy can generate the heating of deep muscle tissue, which simultaneously improves the hemoglobin saturation [5]. The physiological effect of this type of therapy is generated by the application of an electromagnetic field in the human body with a frequency of approximately 0.5 MHz [13]. The effects attributed to this technique include increased deep and superficial blood flow, vasodilation, increased temperature, removal of excess liquid, and increased cell proliferation [14,15].

It has been observed that increase in blood perfusion is related to increase in temperature, but others, like cell proliferation, seem to be primarily related to current flow [14,15]. It has been proven that cell proliferation begins at 0.00005 A per square millimeter of current flow [15].

There are clinical publications that support the use of CRet therapy, although the amounts of energy and current that must be transferred to obtain the desired changes in temperature and current flow are unknown. Furthermore, the control of these reactions is still highly based on the empirical experience of the therapist [5,16,17,18]. There are only two in vitro studies that analyzed changes in temperature and current flow in cadavers [13,19]. These studies analyzed these variables in the Achilles tendon and myotendinous junction [13] and in the different structures of the knee [19] by applying different capacitive and resistive programs at high and low powers for 5 min [13]. Nevertheless, no study to date has studied changes in current flow and temperature when a standardized treatment protocol for chronic elbow tendinopathy is applied. These inconveniences engender the interest in performing in vitro studies to validate the therapeutic effects of frequently applied clinical protocols.

The aim of this in vitro study was to analyze the thermal behavior and transmission of electric current in the superficial and deep tissues of the elbow during the application of different modalities of a CRet treatment protocol for chronic elbow tendinopathy.

## 2. Materials and Methods

### 2.1. Study Design

An experimental in vitro study was designed to determine the effect of a CRet treatment protocol for chronic elbow tendinopathy, using the “Wintecare T-Plus” device, on temperature and electric current. The body donation program of the Facultad de Medicina y Ciencias de la Salud de la Universitat Internacional de Catalunya provided all the cadavers. This study was approved by the ethics committee “Comitè d’Ètica de Recerca (CER) de la Universitat Internacional de Catalunya” with reference number CBAS-2019-18.

### 2.2. Anatomic Sample

The anatomic sample was composed of 10 elbows from 5 cryopreserved full cadavers (3 men and 2 women). The corpses were stored at −16 °C and were kept at room temperature for 48 h until the day of the study. The mean age of the cadavers was 80.6 ± 14.6. None of the cadaveric specimens used for this study had evidence of traumatic injuries or surgical scars in the upper extremities [20].

### 2.3. Intervention

**Figure 1:**
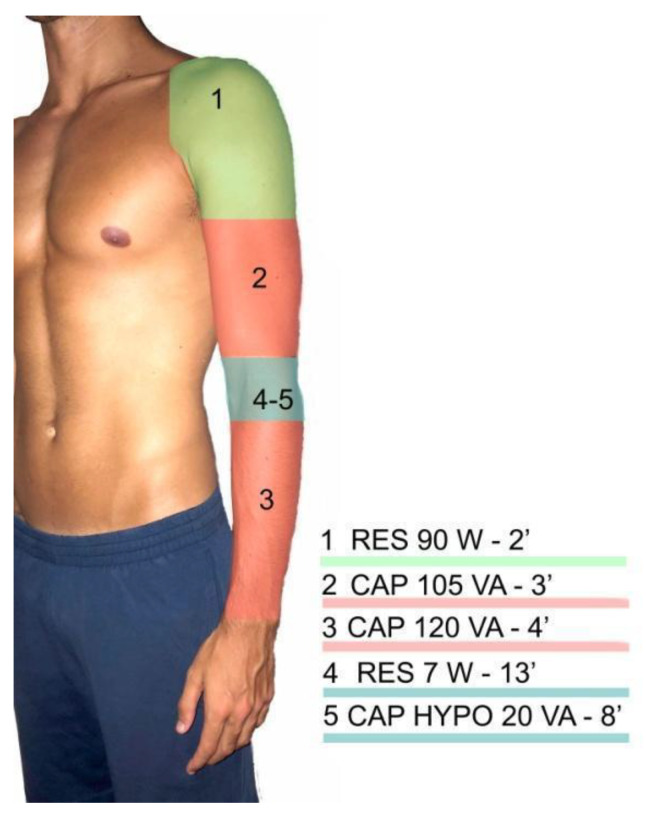
Applications used during the protocol. RES, resistive; W, watts; CAP, capacitive; VA, volt-amperes; CAP HYPO, hypothermic capacitive.

A 30 min intervention was performed based on a CRet protocol commonly used in clinics for the treatment of chronic lateral elbow tendinopathy (Figure 1). Throughout the whole treatment, the therapist could identify and control the power level used during the therapy and the watts (the absorbed power) shown by the device during the application. A T-Plus device application range varies from 1 to 300 watts in the resistive mode and from 1 to 450 VA in the capacitive mode [13].

The protocol consists of different applications. During the first 2 min, a resistive application of 90 W in the shoulder region was applied. This application was made with the clinical aim of generating an increase in blood perfusion. In the next 3 min, a capacitive application of 105 VA within the bicep brachialis region was applied. The clinical aim of this application was to generate vasodilation [13]. Afterwards, a capacitive application of 120 VA was applied for 4 min. In this case, the aim was to increase the temperature in the muscle bellies of the wrist extensors [13]. Subsequently, the main treatment was applied in the lateral epicondyle region of the elbow with a resistive approach of 7 W [13]. The purpose of this part of the protocol was to repair the tissue through cell proliferation caused by the current flow. Finally, a capacitive application of 20 VA was implemented for 8 min in the elbow region with a hypothermic electrode. The objective of this last application was to normalize the temperature and generate tissue drainage.

Dynamic movements were made, similar to those applied in real patients, with a constant pressure. The treatments were made by a physiotherapist with experience in the utilization of the T-Plus.

### 2.4. Experimental Procedure

Each cadaver was placed in supine position with their forearms in pronation, slight elbow flexion, and neutral shoulder flexion/extension.

The order of the protocol application on the arms of each cadaver was randomly assigned prior to the start of the study. This randomization was done by an external investigator using the web program “random.org.” Before the treatment application, it was ensured that each cadaver’s basal temperature was stable.

**Figure 2:**
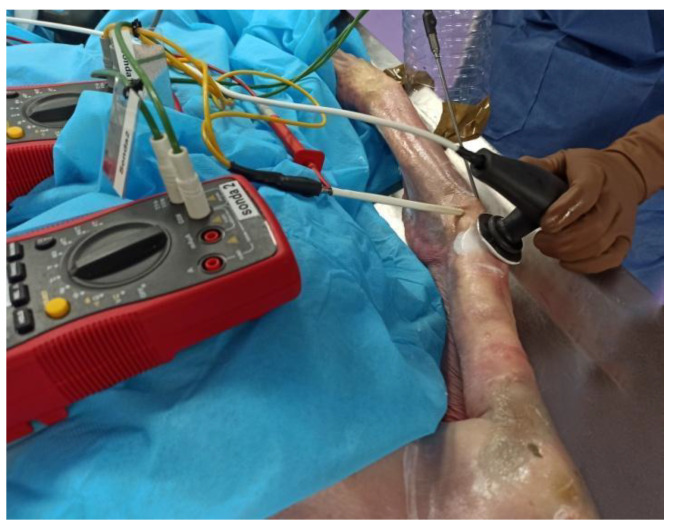
Invasive placement of the thermocouples in the common extensor tendon and the radiohumeral capsule.

All of the instrumentations used in this study possessed a calibration certificate. “Hart Scientific PT25 5628-15” thermocouples were employed to measure the temperature (°C) of the common extensor tendon in its proximal insertion and the radiohumeral capsule (Figure 2). A “Thermocomed” digital thermometer was used to measure the surface temperature of the elbow region. The invasive thermocouples were set by an investigator with sonographic experience with the help of a “U.S. Aloka Prosound C3 15.4” ultrasound with a high-frequency linear transducer (USTTL01, 12L5).

The return electrode of the CRet equipment was placed in the lumbar region of the cadavers. The treatment was performed using the hand electrode of the CRet equipment according to the previously described treatment protocol. The initial superficial temperature and the common extensor and radiohumeral capsule temperatures were measured to establish a baseline. These measurements were also registered after each application for the duration of the 30 min treatment protocol. Before the treatment, the impedance (Multimeter Fluke 8846A) was always measured to guarantee that the values shown by the T-Plus Wintecare device were correct. Furthermore, the current flow was calculated in each application using the average voltage divided by the initial impedance.

### 2.5. Statistical Analysis

The statistical analysis was realized by using the SPSS Statistics software (IBM, Armonk, NY, USA) for Windows. The normality of the distribution was calculated using the Shapiro–Wilk test (*p* > 0.05). The mean, standard deviation, and percentage of change in the superficial, common extensor tendon, and radiohumeral capsule temperatures at the end of each part of the protocol were measured.

The differences between the application moments were calculated using the Wilcoxon test. A *p* < 0.05 value was considered statistically significant.

## 3. Results

**Figure 3:**
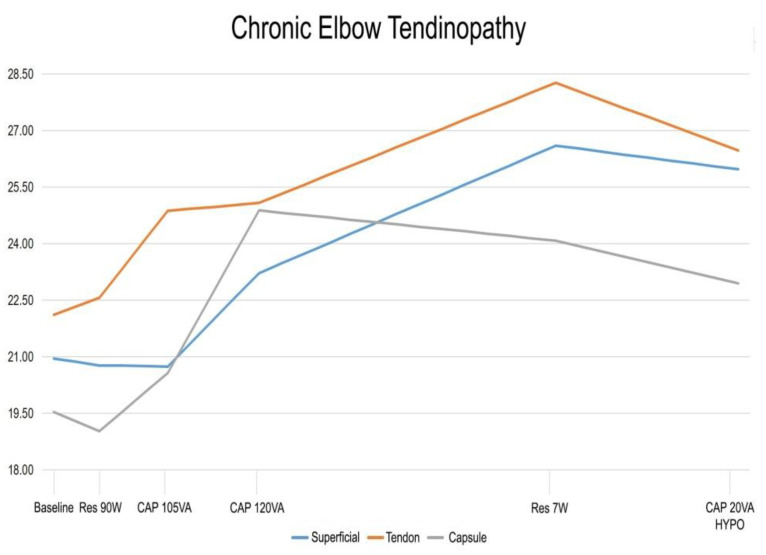
Graphic of the temperature variation during the whole treatment protocol. RES, resistive; W, watts; CAP, capacitive; VA, volt-amperes; CAP HYPO, hypothermic capacitive.

Descriptive outcomes in the common extensor tendon and radiohumeral joint capsule are shown in Table 1 and Figure 3.

The current flows during the different parts of the protocol were 0.34 ± 0.22 A (RES 90 W), 0.14 ± 0.57 A (CAP 105 VA), 0.17 ± 0.46 A (CAP 120 VA), 0.24 ± 0.11 A (RES 7 W), and 0.12 ± 0.03 A (CAP 20 VA).

The application showed a statistically significant increase in temperature in all the evaluated points at the end of the 30 min protocol compared with the baseline temperature. The superficial temperature increased by 24% (5.02°) (*p* < 0.005), the common extensor tendon by 19.7% (4.36°) (*p* < 0.007), and the radiohumeral joint capsule by 17.5% (3.41°) (*p* < 0.005).

In the first two applications, the superficial temperature presented a slight drop of 0.18° and a progressive increase during the next two applications. The CAP 120 VA application was the only application that obtained a statistically significant difference (*p* < 0.005) compared with the baseline value with an increase of 2.48° of the superficial temperature. During the application of CAP 20 VA, a nonsignificant decrease in temperature of 0.63° was observed.

The temperature of the common extensor tendon increased during the duration of the protocol, except for the last application (CAP 20 VA), which registered a drop of 1.80°, reaching a statistically significant difference (*p* < 0.007). The other applications of the protocol achieved an increase between 0.21° and 3.19°. A statistically significant difference was attained with the RES 90 W application (0.45°, *p* < 0.007) and CAP 105 VA (2.31°, *p* < 0.011).

The radiohumeral capsule showed a slight decrease in the RES 90 W application from the baseline temperature, with a 0.51° drop, that did not reach a statistically significant difference (*p* < 0.476). A drop in temperature was also observed in the last two applications, RES 7 W with a statistically insignificant decrease of 0.80° (*p* < 0.386) and CAP 20 VA with a statistically significant drop of 1.14° (*p* < 0.005). A rise in temperature was found during the CAP 105 VA (1.55°) and CAP 120 V (4.31°) applications. These applications reached statistically significant differences of *p* < 0.019 and *p* < 0.028, respectively.

In Figure 3, the progression of the temperature in the different tissues throughout the whole protocol can be observed.

## 4. Discussion

The different applications used during the CRet treatment protocol in this in vitro study produced variable responses and specific effects in temperature and current flow in the common extensor tendon and radiohumeral capsule. As far as the authors know, there are no current studies regarding these variables in the elbow region, neither in cadavers nor in alive subjects.

During all the applications, we observed a current flow of over 0.03 A, which indicates that the application would be capable of generating cell proliferation in the measured structures (extensor tendon of the elbow and radiohumeral capsule) [13,14,15].

The objective of the first application (application 1 in Figure 1—RES 90 W) was to generate a rise in blood perfusion in the adjacent tissues of the elbow. We observed a significant increase in temperature in the common extensor tendon of the elbow despite the application site in the shoulder region. These findings might sustain the theory that this phenomenon happens due to the rise in temperature being directly related to the current flow and increase in blood perfusion because of the Joule effect [21].

During the second application (application 2 in Figure 1—AP 105 VA), the aim was to generate vasodilation in the bicep brachialis region, a structure adjacent to the elbow. We observed a significant rise in temperature at the common extensor tendon and radiohumeral capsule, which is directly related to vasodilation [5,15].

Next, the application on the forearm was carried out (application 3 in Figure 1—CAP 120 VA) with the objective of increasing the temperature and massaging the extensor muscles without influencing the extensor tendon. The objective of this application was to generate an increase in temperature. We observed a significant rise in the superficial and radiohumeral capsule temperatures. Different studies indicate that these types of applications can be useful for the treatment of chronic tendinopathies [13], or fibrosed scars after muscular ruptures, due to the rise in temperature being related to an improvement in tissue viscoelasticity [13,22].

The low-power resistive application (application 4 in Figure 1—RES 7 W) had the longest duration. Its objective was to generate repairment of the affected tissue, so it was directly applied to the lateral epicondyle area. During the application, no significant temperature change was observed, which is a typical finding for low-power applications [13,23]. This application generated the largest current flow with a lower increase in temperature, which is directly related to a rise in cell proliferation and tissue regeneration [13,14,15], a fundamental factor for tendinopathy recovery [13]. Previous studies obtained positive clinical results when resistive and capacitive applications were combined [17,24], similarly to our protocol. However, we cannot compare the obtained results due to the absence of previous bibliography.

The capacitive hypothermic application (application 5 in Figure 1—CAP 20 VA) was the last application. Its goal was to generate drainage and reduce the temperature. Our results showed a significant decrease in temperature in the common extensor tendon of the elbow and radiohumeral capsule. To date, this effect has only been empirically hypothesized, and no study has validated the hypothermic electrode application. Nonetheless, although more research is needed, it seems that these preliminary data could support the clinical reduction of temperature with this application.

## 5. Limitations

This study presents several limitations. First, this study was performed in cadavers, which have no thermoregulation or active blood flow. This factor probably influenced the temperature rises because thermoregulation causes heat dissipation in alive human beings. This effect would help avoid unwanted hyperthermia during treatment in real patients [5]. Another limitation is that even though the cadavers were cryopreserved, the muscle and tendinous properties could vary compared with those of alive subjects. The average age of the used corpses was relatively high. Despite that, the authors considered that the use of the donors’ bodies allowed them to know how a clinically utilized CRet protocol affects the temperature and current flow values in the common extensor tendon and radiohumeral capsule, something that is nonviable in alive subjects. More studies are needed to confirm these effects clinically. There are several CRet protocols for the treatment of lateral elbow pain, so the results of this study cannot be extrapolated to all applications.

## 6. Conclusions

The different applications of the CRet protocol showed specific effects on the temperature and current flow in the common extensor tendon and radiohumeral capsule. All applications of the protocol produced a current flow that is associated with the generation of cell proliferation. The RES 90 W and CAP 105 VA applications significantly increased the temperature of the common extensor tendon. CAP 105 and CAP 120 VA significantly increased the temperature of the radiohumeral capsule. Additionally, CAP 120 VA was the only application that significantly increased the superficial temperature. CAP 20 VA was the only one that generated a significant reduction in the temperatures of the common extensor tendon and radiohumeral capsule.

## Figures and Tables

**Table 1:** Descriptive outcomes of the superficial, common extensor tendon (tendon), and radiohumeral capsule (capsule) temperatures.

	Baseline	RES 90 W 2′	CAP 105 VA 3′	CAP 120 VA 4′	RES 7 W 13′	CAP HYPO 20 VA 8′
Superficial	20.95 ± 1.69	20.77 ± 1.76	20.74 ± 1.73	23.22 ± 3.77	26.60 ± 4.27	25.97 ± 2.00
Tendon	22.11 ± 1.95	22.56 ± 1.78	24.87 ± 6.96	25.08 ± 5.54	28.27 ± 4.99	26,47 ± 2.78
Capsule	19.53 ± 3.18	19.02 ± 2.07	20.57 ± 3.83	24.88 ± 11.44	24.08 ± 3.53	22.94 ± 2.48

Abbreviations: RES, resistive; W, watts; CAP, capacitive; VA, volt-amperes; CAP HYPO, hypothermic capacitive.

## Data Availability

All data regarding this article were uploaded to the Harvard Dataverse and can be found under the DOI 10.7910/DVN/G2GSBX with the title “Replication Data for: CRET on the elbow.”

## References

[1] Shiri R., Viikari-Juntura E., Varonen H., Vaara M.H. (2006). Prevalence and Determinants of Lateral and Medial Epicondylitis: A Population Study. Am. J. Epidemiol..

[2] Swedish Council on Health Technology Assessment (2006). Methods of Treating Chronic Pain: A Systematic Review.

[3] Tarpada S.P., Morris M.T., Lian J., Rashidi S. (2018). Current advances in the treatment of medial and lateral epicondylitis. J. Orthop..

[4] Morishita K., Karasuno H., Yokoi Y., Morozumi K., Ogihara H., Ito T., Fujiwara T., Fujimoto T., Abe K. (2014). Effects of therapeutic ultrasound on intramuscular blood circulation and oxygen dynamics. J. Jpn. Phys. Ther. Assoc. Rigaku Ryoho.

[5] Tashiro Y., Hasegawa S., Yokota Y., Nishiguchi S., Fukutani N., Shirooka H., Tasaka S., Matsushita T., Matsubara K., Nakayama Y. (2017). Effect of Capacitive and Resistive electric transfer on haemoglobin saturation and tissue temperature. Int. J. Hyperth..

[6] Richards H.J. (1980). Repair and healing of the divided digital flexor tendon. Injury.

[7] Fenwick S.A., Hazleman B.L., Riley G.P. (2002). The vasculature and its role in the damaged and healing tendon. Arthritis Res..

[8] Macnab I. (1973). Rotator cuff tendinitis. Ann. R. Coll. Surg. Engl..

[9] Kelly R., Beehn C., Hansford A., Westphal K.A., Halle J.S., Greathouse D.G. (2005). Effect of fluidotherapy on superficial radial nerve conduction and skin temperature. J. Orthop. Sports Phys. Ther..

[10] Halle J.S., Scoville C.R., Greathouse D.G. (1981). Ultrasound’s effect on the conduction latency of the superficial radial nerve in man. Phys. Ther..

[11] Mace T.A., Zhong L., Kokolus K.M., Repasky E.A. (2012). Effector CD8 ^+^ T cell IFN- *γ* production and cytotoxicity are enhanced by mild hyperthermia. Int. J. Hyperth..

[12] Knippertz I., Stein M.F., Dörrie J., Schaft N., Müller I., Deinzer A., Steinkasserer A., Nettelbeck D.M. (2011). Mild hyperthermia enhances human monocyte-derived dendritic cell functions and offers potential for applications in vaccination strategies. Int. J. Hyperth..

[13] López-De-Celis C., Hidalgo-García C., Pérez-Bellmunt A., Fanlo-Mazas P., González-Rueda V., Tricás-Moreno J.M., Ortiz S., Rodríguez-Sanz J. (2020). Thermal and non-thermal effects off capacitive-resistive electric transfer application on the Achilles tendon and musculotendinous junction of the gastrocnemius muscle: A cadaveric study. BMC Musculoskelet. Disord..

[14] Hernández-Bule M.L., Trillo M.Á., Úbeda A. (2014). Molecular mechanisms underlying antiproliferative and differentiating responses of hepatocarcinoma cells to subthermal electric stimulation. PLoS ONE.

[15] Hernández-Bule M.L., Paíno C.L., Trillo M.Á., Úbeda A. (2014). Electric stimulation at 448 kHz promotes proliferation of human mesenchymal stem cells. Cell. Physiol. Biochem..

[16] Yokota Y., Sonoda T., Tashiro Y., Suzuki Y., Kajiwara Y., Zeidan H., Nakayama Y., Kawagoe M., Shimoura K., Tatsumi M. (2018). Effect of Capacitive and Resistive electric transfer on changes in muscle flexibility and lumbopelvic alignment after fatiguing exercise. J. Phys. Ther. Sci..

[17] Muscaritoli M., Molfino A., Lucia S., Fanelli F.R. (2015). Cryoultrasound therapy and tendonitis in athletes: A comparative evaluation versus laser CO2 and t.e.ca.r.therapy. Crit. Rev. Oncol. Hematol..

[18] Takahashi K., Suyama T., Takakura Y., Hirabayashi S., Tsuzuki N., Li Z.-S. (2004). Clinical Effects of Capacitive Electric Transfer Hyperthermia Therapy for Cervico-Omo-Brachial Pain. J. Phys. Ther. Sci..

[19] Rodríguez-Sanz J., Pérez-Bellmunt A., López-de-Celis C., Lucha-López O.M., González-Rueda V., Tricás-Moreno J.M., Simon M., Hidalgo-García C. (2020). Thermal and non-thermal effects of capacitive–resistive electric transfer application on different structures of the knee: A cadaveric study. Sci. Rep..

[20] Pérez-Bellmunt A., Miguel-Pérez M., Brugué M.B., Cabús J.B., Casals M., Martinoli C., Kuisma R. (2015). An anatomical and histological study of the structures surrounding the proximal attachment of the hamstring muscles. Man. Ther..

[21] Grimnes S.M.Ø. (2000). Joule Effect and Temperature Rise.

[22] Habets B., van den Broek A.G., Huisstede B.M.A., Backx F.J.G., van Cingel R.E.H. (2018). Return to Sport in Athletes with Midportion Achilles Tendinopathy: A Qualitative Systematic Review Regarding Definitions and Criteria. Sport. Med..

[23] Li H.Y., Hua Y.H. (2016). Achilles Tendinopathy: Current Concepts about the Basic Science and Clinical Treatments. Biomed Res. Int..

[24] Bito T., Tashiro Y., Suzuki Y., Kajiwara Y., Zeidan H., Kawagoe M., Sonoda T., Nakayama Y., Yokota Y., Shimoura K. (2019). Acute effects of capacitive and resistive electric transfer (CRet) on the Achilles tendon. Electromagn. Biol. Med..

